# Supplementary material for: Comprehensive Analysis of Highbush Blueberry Plants Propagated In Vitro and Conventionally
Source: Int J Mol Sci. 2023 Dec 30;25(1):544. doi: 10.3390/ijms25010544 (PMC10779370; doi:10.3390/ijms25010544)

Highbush blueberries *in vitro* culture axillary shoots derived

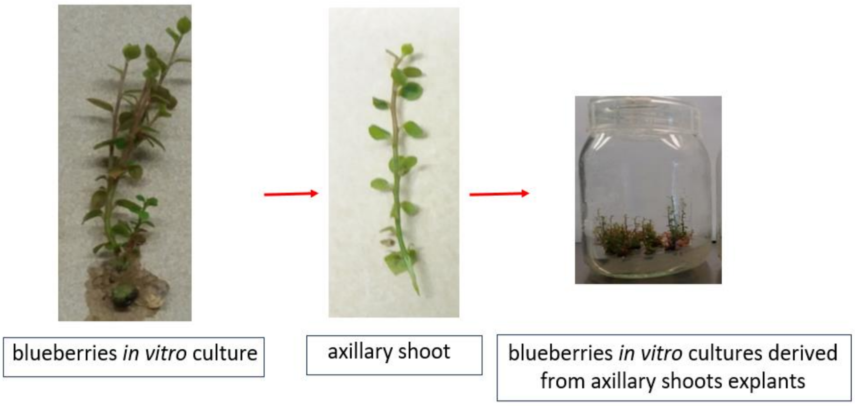

Highbush blueberries *in vitro* culture adventitious shoots derived

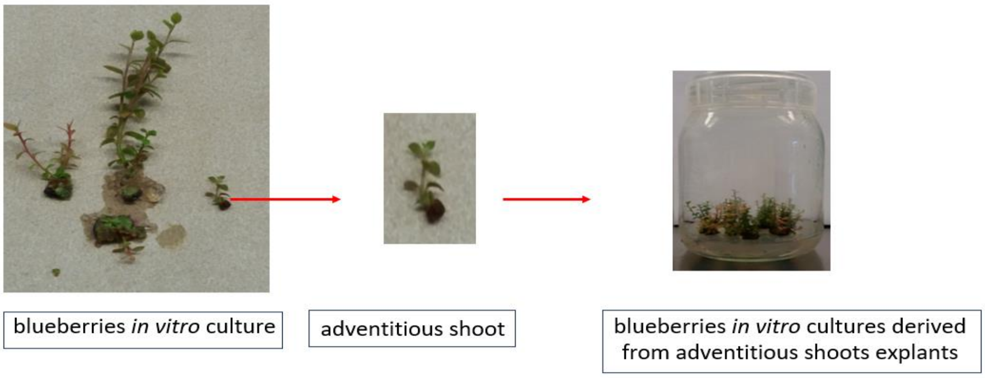

Rooting of 2-node semi-woody cuttings

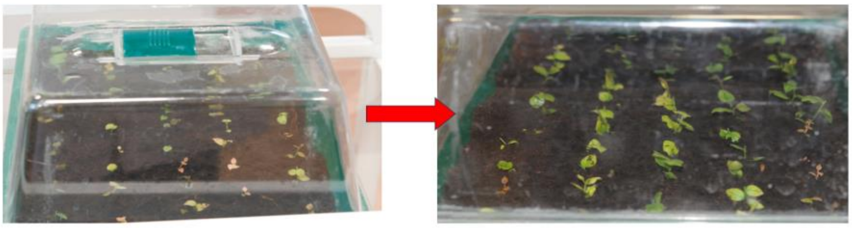

Cultivation of highbush blueberries plants in tops

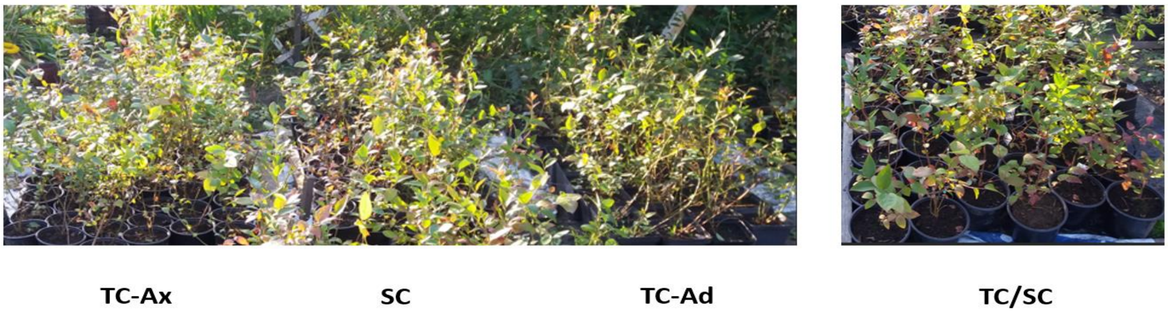

Supplement: Supplementary file 1 [file ijms-25-00544-s001.zip › ijms-2763394-supplementary.pdf]
